# Plants Rather than Mineral Fertilization Shape Microbial Community Structure and Functional Potential in Legacy Contaminated Soil

**DOI:** 10.3389/fmicb.2016.00995

**Published:** 2016-06-24

**Authors:** Jakub Ridl, Michal Kolar, Michal Strejcek, Hynek Strnad, Petr Stursa, Jan Paces, Tomas Macek, Ondrej Uhlik

**Affiliations:** ^1^Department of Genomics and Bioinformatics, Institute of Molecular Genetics, The Czech Academy of Sciences, PragueCzech Republic; ^2^Department of Philosophy and History of Science, Faculty of Science, Charles University in Prague, PragueCzech Republic; ^3^Department of Biochemistry and Microbiology, Faculty of Food and Biochemical Technology, University of Chemistry and Technology, Prague, PragueCzech Republic

**Keywords:** microbial community structure, plants, fertilization, contaminated soil, functional potential

## Abstract

Plant-microbe interactions are of particular importance in polluted soils. This study sought to determine how selected plants (horseradish, black nightshade and tobacco) and NPK mineral fertilization shape the structure of soil microbial communities in legacy contaminated soil and the resultant impact of treatment on the soil microbial community functional potential. To explore these objectives, we combined shotgun metagenomics and 16S rRNA gene amplicon high throughput sequencing with data analysis approaches developed for RNA-seq. We observed that the presence of any of the selected plants rather than fertilization shaped the microbial community structure, and the microbial populations of the root zone of each plant significantly differed from one another and/or from the bulk soil, whereas the effect of the fertilizer proved to be insignificant. When we compared microbial diversity in root zones versus bulk soil, we observed an increase in the relative abundance of Alphaproteobacteria, Betaproteobacteria, Gammaproteobacteria or Bacteroidetes, taxa which are commonly considered copiotrophic. Our results thus align with the theory that fast-growing, copiotrophic, microorganisms which are adapted to ephemeral carbon inputs are enriched in the vegetated soil. Microbial functional potential indicated that some genetic determinants associated with signal transduction mechanisms, defense mechanisms or amino acid transport and metabolism differed significantly among treatments. Genetic determinants of these categories tend to be overrepresented in copiotrophic organisms. The results of our study further elucidate plant-microbe relationships in a contaminated environment with possible implications for the phyto/rhizoremediation of contaminated areas.

## Introduction

Soil as a microbial habitat contains numerous microsites and gradients which harbor enormous microbial diversity. Typically, in a single gram of soil there are billions of microbial cells spanning thousands of microbial species. Rhizosphere, the thin layer of soil directly influenced by the roots, is a particularly active spot for microbial-plant interactions (Chaudhry et al., 2005; Philippot et al., 2013) due to the organic compounds present here that are often lacking in the bulk soil (Hartmann et al., 2009). The main source of plant organic carbon in soil is rhizodeposition, which is the release of compounds from roots into the surrounding soil (Jones et al., 2009; Lambers et al., 2009). Molecules that are rhizodeposited include several sugars, amino acids, organic acids, fatty acids, sterols, growth factors, vitamins, enzymes, flavonoids, nucleotides, plant hormones, alcohols, alkyl sulfides, inorganic ions, and gaseous molecules [for review see Dennis et al. (2010)]. In addition, the activity of roots impacts the physical and chemical conditions of the rhizosphere compartment which, in turn, influences microbial populations. Changes in soil physicochemical conditions can determine the availability and cycling of nutrients including elements/compounds which may be toxic for plants and microorganisms (Neumann et al., 2009). Consequently, a greater density of microorganisms develops in the rhizosphere than bulk soil (Dennis et al., 2010).

In polluted soils, rhizosphere interactions are of particular importance and much research have been devoted to rhizodegradation (Macek et al., 2000; Singer et al., 2003; Macková et al., 2006, 2010; Kurzawová et al., 2012), analysis of diversity in polluted rhizospheres (Mukherjee et al., 2014; Stella et al., 2015), as well as linking rhizodegradation potential to microbial diversity (Sipila et al., 2008; Uhlík et al., 2009; Leewis et al., 2016a). First, carbonaceous compounds released into soil either promote microbial cometabolism of many persistent organic pollutants, as is the case with many simple flavonoids (Pham et al., 2012, 2015; Toussaint et al., 2012), or provide carbon and/or energy sources to rhizosphere microbiota (Leigh et al., 2006). Additionally, oxygen content is usually higher in the rhizosphere providing molecules essential for the activity of oxygenases which are often involved in biodegradation processes (Leigh et al., 2002). Furthermore, root exudates also contain biosurfactants that increase the bioavailability of pollutants with low solubility (Read et al., 2003). Research has also shown that plants may promote the mobilization of pollutants into the rhizosphere (Liste and Alexander, 2000; Yi and Crowley, 2007). As a result, the pollutants occur in the rhizosphere at increased concentrations and are more susceptible to biodegradation.

Previous results have shown that horseradish (*A. rusticana*), black nightshade (*S. nigrum*), and tobacco (*N. tabacum*) attenuated polychlorinated biphenyl (PCB) concentrations in legacy contaminated soil (Ionescu et al., 2009; Kurzawová et al., 2012). Based on these studies, we hypothesized that not only do the plants absorb and transform PCBs (Rezek et al., 2008) but also that they alter soil community structure and enrich or stimulate degradative populations. Therefore, we used shotgun metagenomics and 16S rRNA gene amplicon pyrosequencing to determine: (i) the extent to which the presence of a plant shapes bacterial community structure in soil; (ii) how different community structures develop in the root zone of different plant species; (iii) how NPK mineral fertilization affects the structure of the communities; and (iv) what the consequences are of the altered community structure on the level of functional potential in this polluted ecosystem.

## Materials and Methods

### Microcosm Setup

Soil samples were collected from a landfill of legacy contaminated soil in Lhenice, south Bohemia, Czech Republic, from an exposed section of 1 m high soil mound, where the roots of natural vegetation were not visually detected. Agrochemical analyses as well as total content of organic and inorganic pollutants of the soil have been published previously (Pavlíková et al., 2007; Uhlík et al., 2012; Stella et al., 2015). In the currently presented experiment, soil (~350 g) was placed in 1 L incubation pots lined with aluminum foil to prevent sorption of PCBs onto the walls of the pots. Approximately 4 weeks old seedlings of horseradish (*A. rusticana*), black nightshade (*S. nigrum*) or tobacco (*N. tabacum*) were planted in the soil after their roots were washed with tap water to remove any remaining original soil. The plants were incubated in a cultivation room under stable conditions (25°C, 12 h of light a day) for 6 months. The effect of a commercially available NPK mineral fertilizer on soil communities was evaluated in addition to the plant treatments. Microcosms were fertilized with a Univerzal KH fertilizer (Nohel Garden, Czech Republic) of the following percentage content: N 9%, P 4%, K 8%, Mg 3%, trace elements Fe, Zn, Mn, Cu, Mo, B, organic component, and growth hormones. The fertilizer was used according to the manufacturer’s instructions: applied once in 2 weeks with watering, starting dose 15 mL fertilizer per 2 L water, other doses 5 mL fertilizer per 2 L water. Non-vegetated soil (both fertilized and non-fertilized) incubated under the same conditions was used as controls. All microcosms were set up in triplicates. Upon destructive harvesting, soil from each root ball was homogenized and stored at -20°C. DNA was isolated with PowerMax Soil DNA Isolation Kit (Mo Bio Laboratories Inc., USA) using the standard protocol with final concentrating of DNA by gradual ethanol precipitation with glycogen (Uhlík et al., 2009) and pooled from replicate samples for sequencing.

### Shotgun Sequencing

Shotgun libraries for pyrosequencing were prepared according to the Rapid Library Preparation Manual (Roche). Each library was sequenced on one large PicoTiterPlate region using the GS FLX instrument with the Titanium chemistry.

### Shotgun Reads Annotation

The shotgun reads were uploaded to MG-RAST server (Meyer et al., 2008) for quality filtering, dereplication, and automatic annotation. To obtain a taxonomic abundance data set for each sample, we searched the multi-source non-redundant M5NR database under MG-RAST and kept the representative hits with *E*-value ≤ 1e - 5, identity ≥60% and minimum alignment length of 15. The same parameters were applied to create functional profiles searching against the COG database (Tatusov et al., 2000, 2003).

### Comparative Analysis of Metagenomes

To analyze differences between the metagenomes, we adopted an RNA-seq approach using the DESeq2 package (Love et al., 2014) in R statistical software (R Development Core Team, 2009). Briefly, we created contingency tables for different hierarchical levels of taxonomical and functional profiles obtained from MG-RAST and, using the DESeq2 package, we fitted the generalized linear model and searched for pairwise differences between the samples grouped according to fertilization (yes–no) and plant versus plant or plant versus control schemes. Differences were identified as statistically significant if they met two criteria: fold change threshold of 1.2 and false discovery rate cutoff of 0.01; i.e., we kept traits that showed at least 20% difference in one or more pairwise comparisons at the level of 1% chance of false positive identification. To visualize statistically significant differential abundances data were *rlog* normalized and plotted as a heatmap.

### Amplicon Preparation and Sequencing

Genes encoding for 16S rRNA were amplified with primers f563-577: 5′-AYTGGGYDTAAAGNG-3′ (Cole et al., 2009) and r1406-1392: 5′- ACGGGCGGTGTGTRC-3′ (Lane, 1991). Each primer contained a 5′-end sequencing adapter (454 Sequencing Application Brief No. 001-2009, Roche); the forward primer also bore different tags (454 Sequencing Technical Bulletin No. 005-2009, Roche) for different samples. The 20-μL PCR mixture contained 0.2 mM dNTPs (Finnzymes, Finland), 0.25 μM primers (Generi Biotech, Czech Republic), 0.1 mg.mL^-1^ bovine serum albumin (New England BioLabs, Great Britain), 0.4 U of Phusion Hot Start II DNA Polymerase (Finnzymes, Finland) with the corresponding buffer, and template DNA (10–50 ng). The reaction conditions were as follows: 98°C for 30 s, 35 cycles of 98°C for 10 s, 60°C for 30 s, and 72°C for 60 s with final extension at 72°C for 10 min. Obtained PCR products were pooled to approximately the same concentrations of DNA and purified with AMPure XP Beads (Agencourt, Beckman Coulter, USA) following the manufacturer’s instructions in order to remove fragments shorter than 200 bp. Pooled amplicons were sequenced from the forward primer using GS FLX+ chemistry and results were analyzed with gsRunProcessor (Roche).

### Comparative Analysis of Metamicrobiomes

Amplicon data were processed with mothur software package version 1.27 (Schloss et al., 2009). Briefly, (i) the range of flows was set between 650 and 800, (ii) flowgrams were denoised by mothur-implemented translation of PyroNoise algorithm (Quince et al., 2009), (iii) primer sequences and barcodes were trimmed off, (iv) sequences were aligned against the full length SILVA reference alignment (release 119) and filtered to keep sequences minimally 400 bp long, (v) single-linkage pre-clustering was performed allowing one base difference per 100 bp, (vi) chimeric sequences were identified by Perseus (Quince et al., 2011) and were removed from the data set, (vii) singletons and contaminating sequences (i.e., mitochondria, chloroplasts, Eukarya) were removed from the data set; (viii) valid sequences were classified with SILVA full length sequences and taxonomy references (release 119). Finally, the data were *rlog* normalized as described above and metamicrobiomic comparative analysis was performed in the same way as the metagenomic analysis using the adopted RNA-seq approach.

In addition to the sequenced samples, amplicons were prepared from an in-laboratory prepared mock community consisting of the strains *Achromobacter xylosoxidans* A8, *Burkholderia xenovorans* LB400, *Pseudomonas putida* JB, *Rhizobium radiobacter* C58 (*Agrobacterium tumefaciens* C58), *Arthrobacter chlorophenolicus* A6, *Bacillus pumilus* SAFR-032, *Micrococcus luteus* NCTC 2665, and *Rhodococcus jostii* RHA1. The mock community sequences were analyzed in the same manner as the sample sequences as an internal control of the analysis procedure.

Non-metric multidimensional scaling and vector fitting were performed in vegan package (Oksanen et al., 2013) in R statistical software (R Development Core Team, 2009) using *metaMDS* (with arguments *noshare* and *autotransform* set to false) and *envfit* commands, respectively.

### Nucleotide Sequence Accession Numbers

The nucleotide sequences of 16S rRNA from amplicon and shotgun sequencing have been submitted to the Sequence Read Archive (SRA) under the accession numbers ERA168068 and ERA596740.

## Results

### Community Composition

**FIGURE 1:**
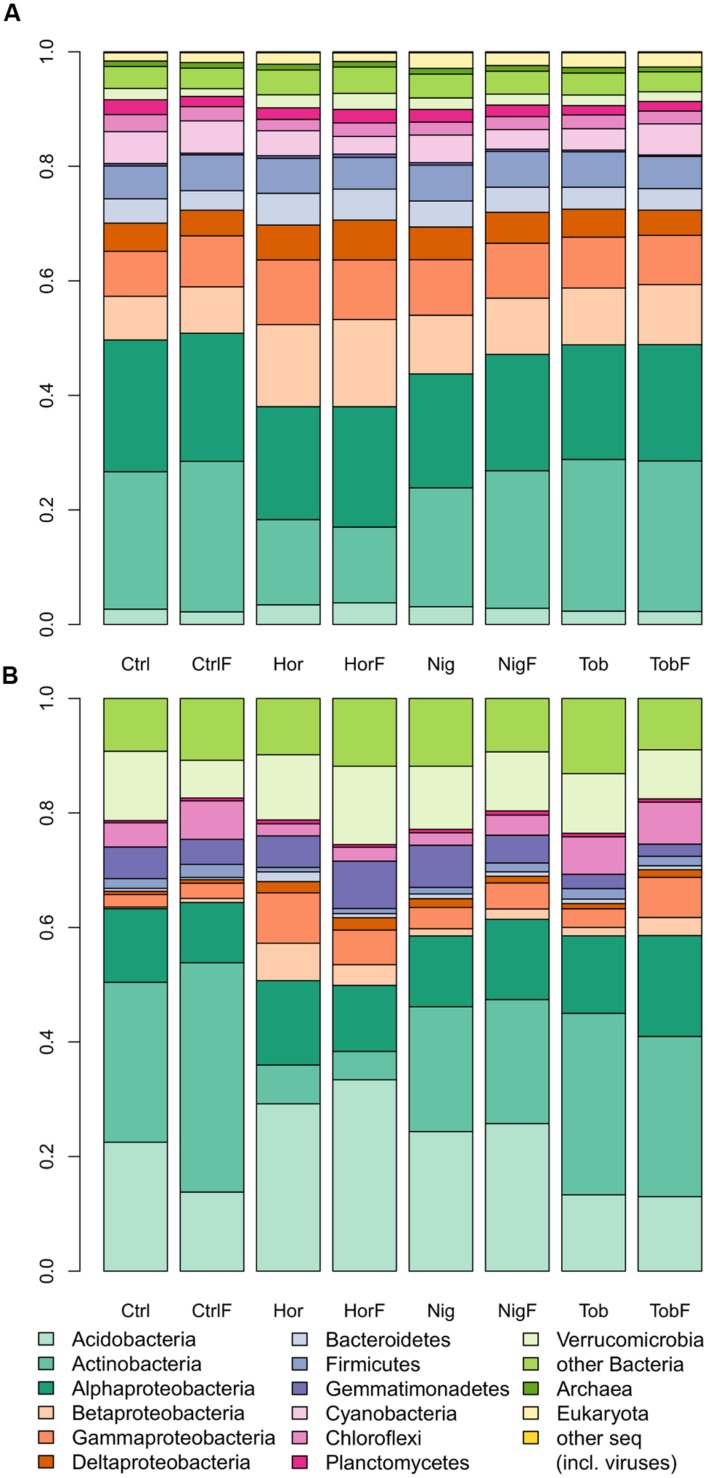
**Community structure in soil samples as determined by shotgun sequencing **(A)** and 16S rRNA gene amplicon analysis (B).** Abbreviations: Ctrl, control soil; CtrlF, fertilized control soil; Hor, horseradish-vegetated soil; HorF, fertilized horseradish-vegetated soil; Nig, nightshade-vegetated soil; NigF, fertilized nightshade-vegetated soil; Tob, tobacco-vegetated soil; TobF, fertilized tobacco-vegetated soil.

**FIGURE 2:**
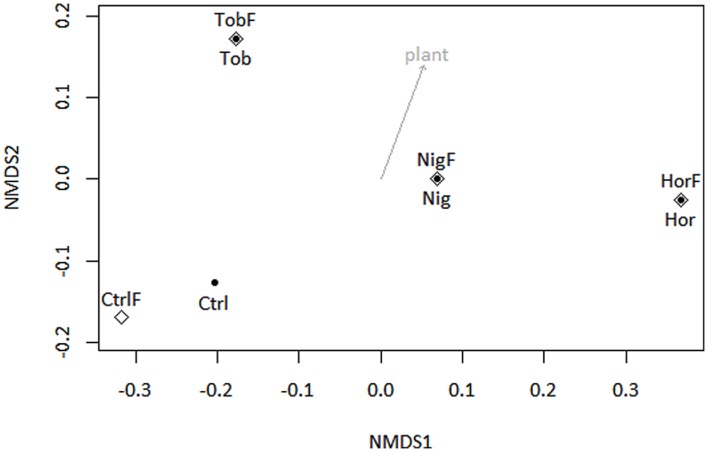
**Non-metric multidimensional scaling ordination analysis (NMDS, stress <0.001) of soil 16S rRNA genes with subsequent fitting of environmental vectors of treatment (plant and fertilizer) onto the ordination (*P* < 0.05, 40,320 permutations).** Abbreviations correspond to those in **Figure 1**. Symbols: full points – non-fertilized treatments, open diamonds – fertilized treatments.

Microbial community composition was determined by taxonomic assignment of shotgun reads. The shotgun metagenomic data indicated that sequenced metagenomes were almost entirely composed of bacterial genomes, with 96.79 ± 0.46% (mean ± SD, **Figure 1A**) of reads being classified as derived from bacterial DNA. Therefore, sequence analysis of 16S rRNA gene pyrotags amplified from soil metagenomes was performed in support of the shotgun metagenomics data. The 16S rRNA gene analysis (**Figure 1B**) indicated that bacterial community structure was dominated by reads affiliated to Actinobacteria in bulk soil and tobacco-vegetated soil. In the horseradish- and nightshade-vegetated soils, the majority of reads clustered with Acidobacteria, with the difference being more pronounced in the horseradish-vegetated samples. Proteobacteria were in all cases dominated by the Alphaproteobacterial class. Beta-, Gamma-, and Deltaproteobacteria were enriched in all vegetated soils. The presence of a plant seemed to shape the bacterial community structure rather than fertilization (NMDS based on Bray–Curtis measure of dissimilarity, **Figure 2**). Vector fitting indicated that the presence of plants explained a significant portion of variation in bacterial community structure (*P* < 0.05), whereas fertilization was not statistically significant. Taxonomic diversity in the vegetated soils was weakly, but consistently, higher compared to bulk soil, and differences in Simpson indices were marginally significant (Mann–Whitney test, *P* = 0.07, **Supplementary Table S1**).

### Who Makes the Difference?

**FIGURE 3:**
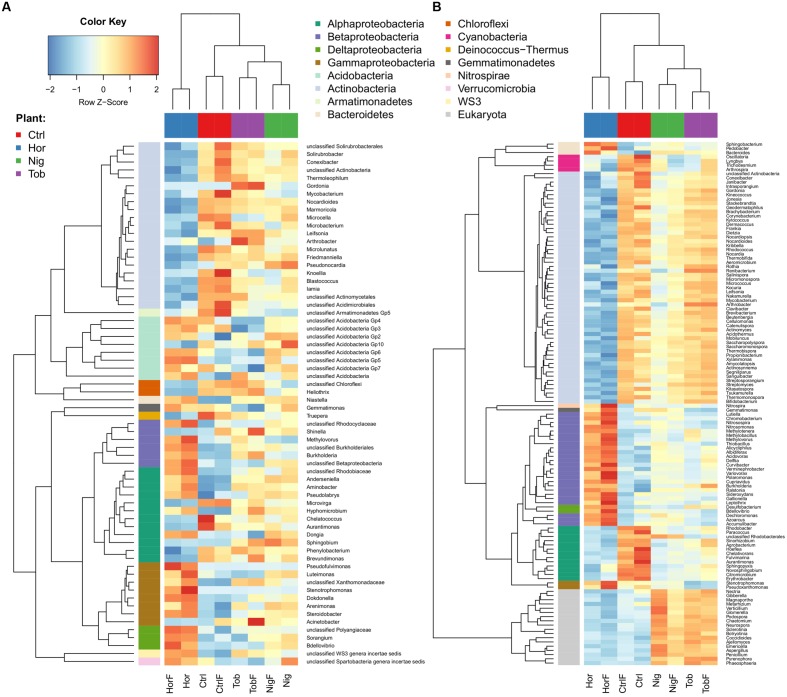
**Differentially abundant taxa in soil samples as determined by 16S rRNA gene amplicon analysis **(A)** and shotgun taxonomical data (B).** Abbreviations correspond to those in **Figure 1**. Each row corresponds to one taxon. To improve readability, the respective *rlog*-normalized read counts were scaled to have zero mean μ and unit standard deviation σ. Thus, dark blue (or red) denotes decrease (or increase) of the *rlog*-normalized counts by 2σ from the mean μ. Yellow denotes average number of counts μ.

We determined if microbial guilds were differentially represented through the identification of pairwise differences between the data grouped according to fertilization (yes–no) and plant–plant or plant-control schemes. Differences between treatments are depicted in **Figure 3**, with the heatmap displaying amplicon data (**Figure 3A**) showing similar trends as the shotgun taxonomic data (**Figure 3B**), which also include the sequences from fungal populations. The communities established in the horseradish root zone differed the most from any other treatment and contained less Actinobacteria, including the genus *Streptomyces*, and more Gammaproteobacteria, except *Acinetobacter*, Deltaproteobacteria as well as Betaproteobacteria, including the genus *Burkholderia*. Actinobacterial genera *Arthrobacter* and *Gordonia* were better represented in the tobacco root zone. Non-vegetated (control) soil was richer in some Actinobacteria and less represented by Betaproteobacteria (**Figure 3**). In horseradish-vegetated and bulk soil, fungal sequences were a significantly smaller portion of the community compared to soils vegetated with nightshade or tobacco (**Figure 3B**).

### Functional Potential

The functional potential of communities was determined by affiliating shotgun data into COG categories as depicted in **Supplementary Figure S1**. This analysis indicated that the metagenomes were very similar in terms of functional potential, being dominated by reads affiliated to COG categories *General function prediction only*, followed by *Amino acid transport and metabolism*; *Energy production and conversion*; *Signal transduction mechanisms*; *Replication, recombination and repair*; *Carbohydrate transport and metabolism*; *Translation, ribosomal structure and biogenesis* and others.

### What Makes the Difference?

**FIGURE 4:**
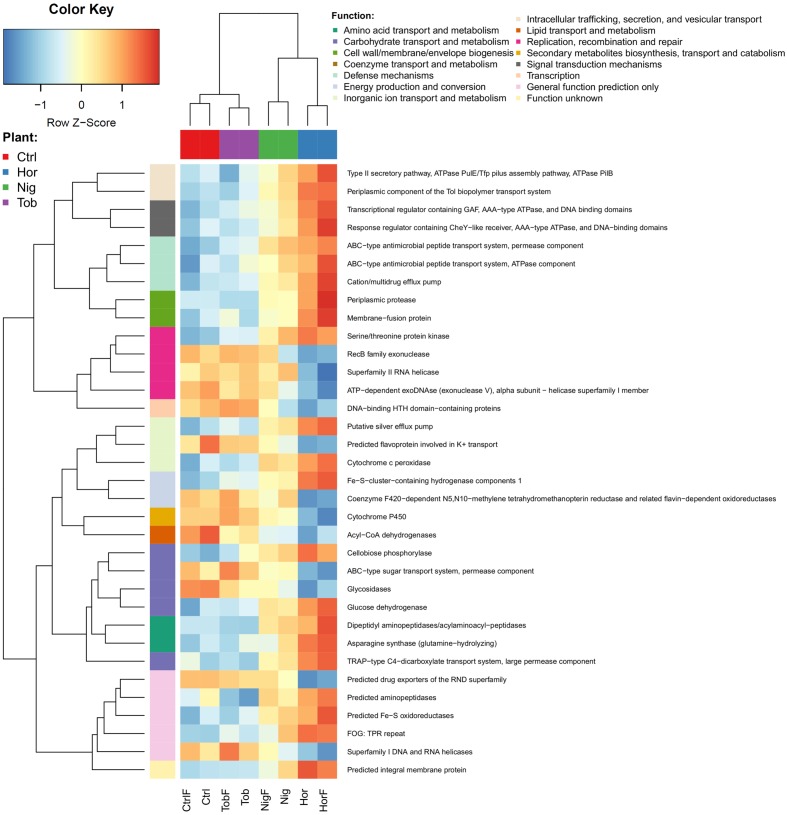
**Differences in the functional potential in the soil samples as determined by annotating metagenomic reads using COG database Level 3.** Abbreviations correspond to those in **Figure 1**. As in **Figure 3**, rlog-normalized read counts of each functional group were scaled to have zero mean and unit standard deviation.

Similar to taxonomic data, the functional potential data showed fewer differences between the fertilized and non-fertilized treatments than between different plants as well as plants and bulk soil. When using COG categories at level 2, only two categories proved to significantly differ between the treatments – *Defense mechanism* and *Cytoskeleton*. Both of these categories had a lower representation in the bulk soil than in the vegetated soils. Many more differences between the treatments were found on the level of COG function (level 3), where specific functions from 14 categories were identified (**Figure 4**), with the profile associated with tobacco plants being the most similar to the control. Statistically significant differences in individual COGs are visualized in the **Supplementary Figure S2**.

## Discussion

In this study, we sought to better understand the role of vegetation and fertilization in shaping microbial community structure and functional potential in legacy contaminated soil. Our approach included a comparative analysis of both shotgun metagenomic and amplicon data sets using an adopted RNA-seq approach, which was originally designed to estimate differences in gene expression using the data from cDNA sequencing (Love et al., 2014), but appears equally beneficial for unraveling differential abundance of OTUs, species counts and functional systems in metagenomic shotgun and amplicon reads. Therefore, we were able to analyze all of our data sets, the shotgun taxonomical and functional profiles, as well as the 16S rRNA amplicon databases, using the same methods. Additionally, it has been previously suggested that, based on simulated and empirical data, the RNA-seq approach is better suited for metagenomic analyses toward normalization and generalized linear model fitting than the widely used methods relying on rarefying, i.e., data normalization by sub-sampling to the smallest sample size (McMurdie and Holmes, 2014).

Metagenomic analysis showed that the samples were strongly dominated by bacterial genomes (**Figure 1A**), which is in accordance with previous quantification of biomass in the same soil; Stella et al. (2015) showed that PLFA specific for bacteria were two orders of magnitude more abundant than fungi. Therefore, the amplicon sequencing targeted bacterial taxa. The major advantage of shotgun metagenomics in comparison to amplicon sequencing is that there are no biases associated with the amplification of 16S rRNA genes. However, there are other potential issues associated with shotgun metagenomics, including lack of sequencing depth to target low-abundance taxa and references being biased toward bacterial lineages which are easily cultured (Shah et al., 2012). Combining both approaches, such as in this study, appears to allow us to answer important microbial ecological questions even though rarefaction curves derived from both amplicon and shotgun data (**Supplementary Figure S3**) indicated enormous phylogenetic diversity in the investigated soil. Overall, the major bacterial groups detected from shotgun data were Actinobacteria and Alphaproteobacteria, followed by other Proteobacteria and Firmicutes (**Figure 1A**). The 16S rRNA gene amplicon data (**Figure 1B**) gave different results, with a lower relative abundance of Alphaproteobacteria and Firmicutes, no detection of Cyanobacteria and higher relative abundance of Acidobacteria. The lower proportion of reads affiliated to Alphaproteobacteria and Cyanobacteria in the amplicon data versus shotgun data was not surprising as the mitochondrion and chloroplast, which are phylogenetic descendants of Alphaproteobacteria and Cyanobacteria, respectively, are commonly contained in the metagenome (Ni et al., 2013). Consequently, some metagenome shotgun reads are derived from these organelle genes, whereas amplicon data are treated to remove such sequences as contaminants from analyzed reads. Lower amounts of Acidobacteria-affiliated reads in the shotgun data, on the other hand, can be ascribed to the lack of information on genetic determinants of this phylum (Lee et al., 2015). For these reasons, the 16S rRNA gene amplicon data seem to provide a better and more precise insight into bacterial community diversity.

### Bulk versus Vegetated Soil

Studying microbial communities influenced by vegetation has long been the main focus of research (Smalla et al., 2001; Kowalchuk et al., 2002; Sipila et al., 2008; Berg and Smalla, 2009). In vegetated systems, bacterial diversity tends to be higher in the bulk soil than rhizosphere, whereas bacterial cell density is higher in the rhizosphere (Dennis et al., 2010; Philippot et al., 2013). In our samples, Simpson indices indicated that alpha diversity increased in the presence of plants, yet with marginal significance (*P* = 0.07). It is important to note that our soil was very different from those traditionally studied from the agricultural point of view, such as grassland soils – it was a sandy loam, very poor in nutrients and organic matter, and furthermore contaminated with PCBs and high levels of chromium and zinc (Stella et al., 2015). Detecting increased diversity in our root zones may indicate that vegetated soil is an area with potentially more efficient contaminant removal from the soil, which is in accordance with previous studies (Macková et al., 2006).

We also found that reads affiliated with some genera of previously described PCB-degraders were more abundant in vegetated soils versus bulk soil, which may indicate the potential for more efficient degradation. For instance, planting with horseradish resulted in an increased abundance of *Burkholderia*, which have thoroughly been described as very efficient degraders of PCBs (Mukerjee-Dhar et al., 1998; Tillmann et al., 2005; Chain et al., 2006; Uhlík et al., 2013), *Stenotrophomonas*, which has been isolated from the same soil by growth on biphenyl (Uhlík et al., 2013), or *Methylovorus*, which has been directly implicated in biphenyl metabolism through stable isotope probing in the horseradish rhizosphere (Uhlík et al., 2009). We also determined that horseradish-vegetated soil had the most unique soil microbial community when compared to the control and other two plant samples (**Figure 3**). *Arthrobacter* (Gilbert and Crowley, 1997; Abraham et al., 2005; Leigh et al., 2007) and *Gordonia* (Koubek et al., 2012), also often associated with PCB-degradation, were significantly enriched in soils vegetated by tobacco.

### Plant versus Fertilizer

Due to the importance of fertilization for agricultural and environmental sciences, assessment of the effects of chemical fertilization is an active field of study (Geisseler and Scow, 2014), especially in connection with attribution of soil functions to specific microbial populations (Su et al., 2015). Many studies have demonstrated that there are shifts in soil microbial community structure associated with the use of chemical fertilizers (Leff et al., 2015; Su et al., 2015) but the populations are less strongly influenced by the fertilization than plant species (Benizri and Amiaud, 2005; Liliensiek et al., 2012), sometimes indicating the fertilizer does not have a significant effect (Marschner et al., 2001; Liliensiek et al., 2012). Our results also indicate that the response of soil microbial populations, in terms of both phylogeny and functional potential, to fertilization is not significant, however, the presence of plants is (**Figures 2**–**4**).

### Copiotrophs versus Oligotrophs

Soils tend to select for copiotrophic or oligotrophic microbial populations based on the amount of available organic carbon (Fierer et al., 2007). Therefore, soils directly under the influence of plants tend to favor copiotrophic microorganisms in comparison to populations in bulk soil (Dennis et al., 2010). Although the ecological grow strategies cannot be completely generalized on the level of phylum or class, bacteria affiliated with Actinobacteria, Bacteroidetes, Alphaproteobacteria, Betaproteobacteria, and Gammaproteobacteria are commonly considered copiotrophic whereas Acidobacteria or Planctomycetes oligotrophic (Fierer et al., 2007; Prober et al., 2015; Leewis et al., 2016b). Increases in the relative abundances of Alpha-, Beta-, Gammaproteobacteria, or Bacteroidetes (**Figure 1**) in vegetated soils also supports the hypothesis that the vegetated soil was enriched for copiotrophic organisms. The trend with Acidobacteria and Planctomycetes, commonly considered oligotrophic, was not that unambiguous. Whereas Planctomycetes were of low abundance throughout the treatments, Acidobacteria were dramatically differently represented, and were enriched especially in the root zone of horseradish. Previously, spatial distributions of groups of Acidobacteria were found to be pH-specific (Mukherjee et al., 2014), and plant-specific colonization of rhizosphere was proposed for certain acidobacterial lineages (Nunes da Rocha et al., 2013). Our results suggest that horseradish creates favorable environment for Acidobacteria, especially Gp4 and Gp6, which were previously found among those favoring higher pH (Mukherjee et al., 2014).

Importantly, several functional categories have also been attributed to either copiotrophic or oligotrophic strategies. We observed that some genetic determinants associated with signal transduction mechanisms, defense mechanisms or amino acid transport and metabolism, categories which tend to be overrepresented in copiotrophic organisms (Lauro et al., 2009; Leff et al., 2015), significantly differed among treatments, with significantly lower abundances in the bulk soil compared to vegetated soils (**Figure 4**).

## Conclusion

In summary, our study indicates that plants, and not fertilization, are the features which drive microbial community structure in this contaminated soil, with the magnitude of the effect depending on the plant species. We also demonstrate that vegetated soils favor copiotrophic bacterial taxa. This information can help us to better understand the plant-microbe relationships in contaminated environments, therefore allowing us to better understand the complicated dynamics potentially associated with the phyto/rhizoremediation of contaminated areas.

## Author Contributions

Conceived and designed the experiments: JP and TM. Performed the experiments: JR, MS, and PS. Analyzed the data: JR, MK, MS, HS, and OU. Contributed reagents/materials/analysis tools: MK, JP, HS, and TM. Wrote the paper: JR, MK, MS, and OU.

## Conflict of Interest Statement

The authors declare that the research was conducted in the absence of any commercial or financial relationships that could be construed as a potential conflict of interest.

## Supplementary Material

FIGURE S1 **Overview of relative abundance of shotgun reads affiliated to COG categories**. Abbreviations correspond to those in **Figure 1**.

FIGURE S2 **Differences in the functional potential in the soil samples as determined by annotating metagenomic reads using individual COGs**. Abbreviations correspond to those in **Figure 1**. As in **Figure 3**, *rlog*-normalized read counts of each functional group were scaled to have zero mean and unit standard deviation.

FIGURE S3 **Rarefaction curves for 16S rRNA gene amplicon and shotgun data**. Abbreviations correspond to those in **Figure 1**.

TABLE S1 **Simpson diversity indices of microbial communities based on 16S rRNA gene amplicon data and a box plot showing fertilized and non-fertilized treatments grouped together**. Abbreviations correspond to those in **Figure 1**.
