# Supplementary material for: Plants Rather than Mineral Fertilization Shape Microbial Community Structure and Functional Potential in Legacy Contaminated Soil
Source: Front Microbiol. 2016 Jun 24;7:995. doi: 10.3389/fmicb.2016.00995 (PMC4919359; doi:10.3389/fmicb.2016.00995)
Supplement: FIGURE S1 — Overview of relative abundance of shotgun reads affiliated to COG categories. Abbreviations correspond to those in Figure 1. [file Data_Sheet_1.ZIP › Supplementary Figure 1.pdf]

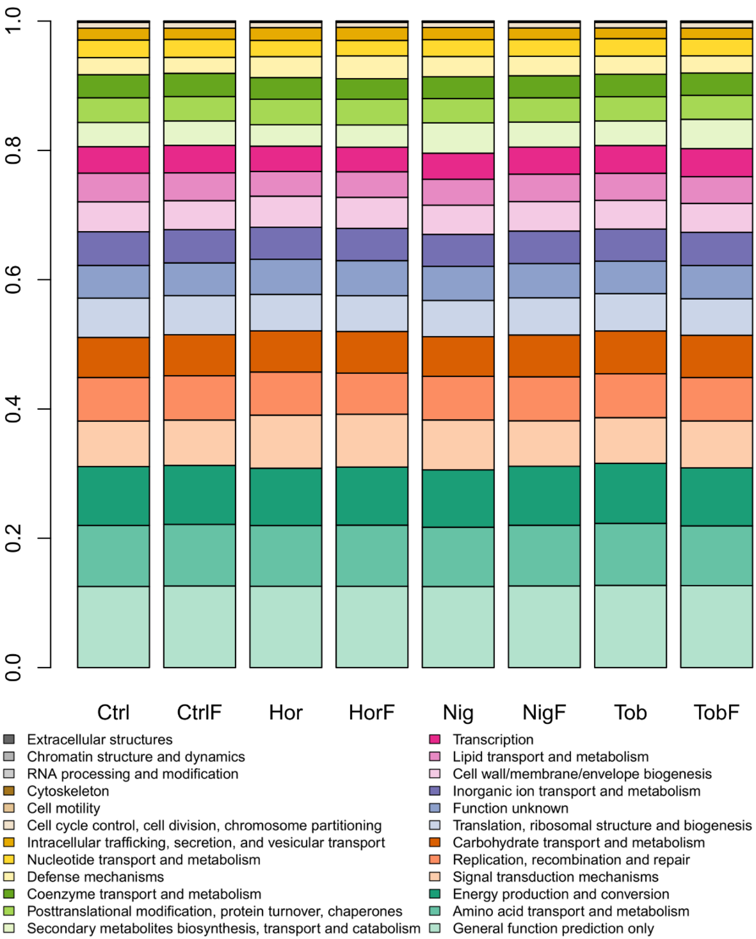
1.0
0.8
0.6
0.4
0.2
0.0
Ctrl
CtrlF
Hor
HorF
Nig
NigF
Tob
TobF
Extracellular structures
Chromatin structure and dynamics
RNA processing and modification
Cytoskeleton
Cell motility
Cell cycle control, cell division, chromosome partitioning
Intracellular trafficking, secretion, and vesicular transport
Nucleotide transport and metabolism
Defense mechanisms
Coenzyme transport and metabolism
Posttranslational modification, protein turnover, chaperones
Secondary metabolites biosynthesis, transport and catabolism
Transcription
Lipid transport and metabolism
Cell wall/membrane/envelope biogenesis
Inorganic ion transport and metabolism
Function unknown
Translation, ribosomal structure and biogenesis
Carbohydrate transport and metabolism
Replication, recombination and repair
Signal transduction mechanisms
Energy production and conversion
Amino acid transport and metabolism
General function prediction only
