# Supplementary material for: Plants Rather than Mineral Fertilization Shape Microbial Community Structure and Functional Potential in Legacy Contaminated Soil
Source: Front Microbiol. 2016 Jun 24;7:995. doi: 10.3389/fmicb.2016.00995 (PMC4919359; doi:10.3389/fmicb.2016.00995)
Supplement: FIGURE S1 — Overview of relative abundance of shotgun reads affiliated to COG categories. Abbreviations correspond to those in Figure 1. [file Data_Sheet_1.ZIP › Supplementary Figure 2.pdf]

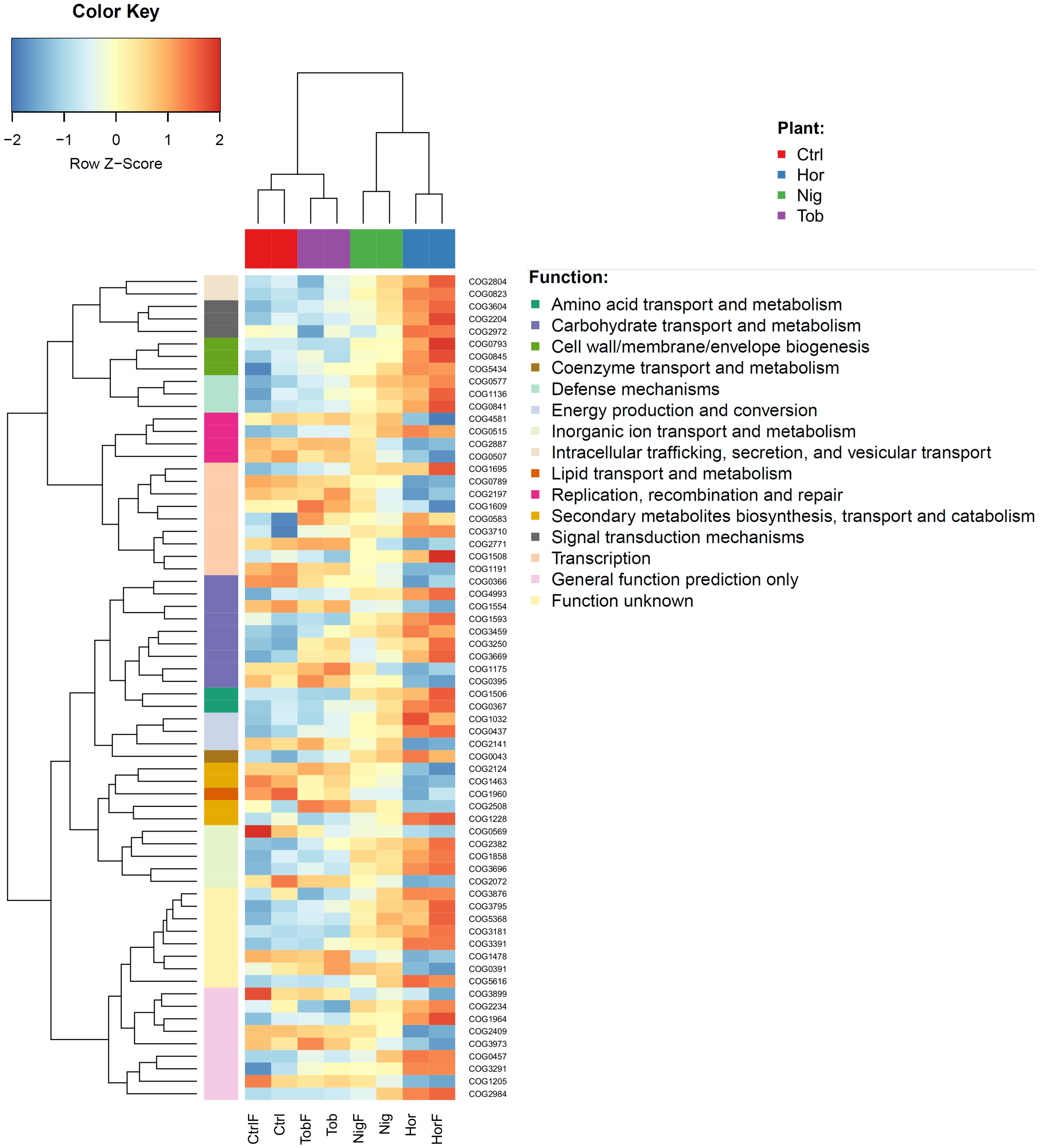

Color Key
−2 −1 0 1 2
Row Z−Score
Plant:
Ctrl
Hor
Nig
Tob
Function:
Amino acid transport and metabolism
Carbohydrate transport and metabolism
Cell wall/membrane/envelope biogenesis
Coenzyme transport and metabolism
Defense mechanisms
Energy production and conversion
Inorganic ion transport and metabolism
Intracellular trafficking, secretion, and vesicular transport
Lipid transport and metabolism
Replication, recombination and repair
Secondary metabolites biosynthesis, transport and catabolism
Signal transduction mechanisms
Transcription
General function prediction only
Function unknown
COG2804
COG0823
COG3604
COG2204
COG2972
COG0793
COG0845
COG5434
COG0577
COG1136
COG0841
COG4581
COG0515
COG2887
COG0507
COG1695
COG0789
COG2197
COG1609
COG0583
COG3710
COG2771
COG1508
COG1191
COG0366
COG4993
COG1554
COG1593
COG3459
COG3250
COG3669
COG1175
COG0395
COG1506
COG0367
COG1032
COG0437
COG2141
COG0043
COG2124
COG1463
COG1960
COG2508
COG1228
COG0569
COG2382
COG1858
COG3696
COG2072
COG3876
COG3795
COG5368
COG3181
COG3391
COG1478
COG0391
COG5616
COG3899
COG2234
COG1964
COG2409
COG3973
COG0457
COG3291
COG1205
COG2984
CtrlF
Ctrl
TobF
Tob
NigF
Nig
Hor
HorF
