# Supplementary figures and images for: Plants Rather than Mineral Fertilization Shape Microbial Community Structure and Functional Potential in Legacy Contaminated Soil
Source: Front Microbiol. 2016 Jun 24;7:995. doi: 10.3389/fmicb.2016.00995 (PMC4919359; doi:10.3389/fmicb.2016.00995)

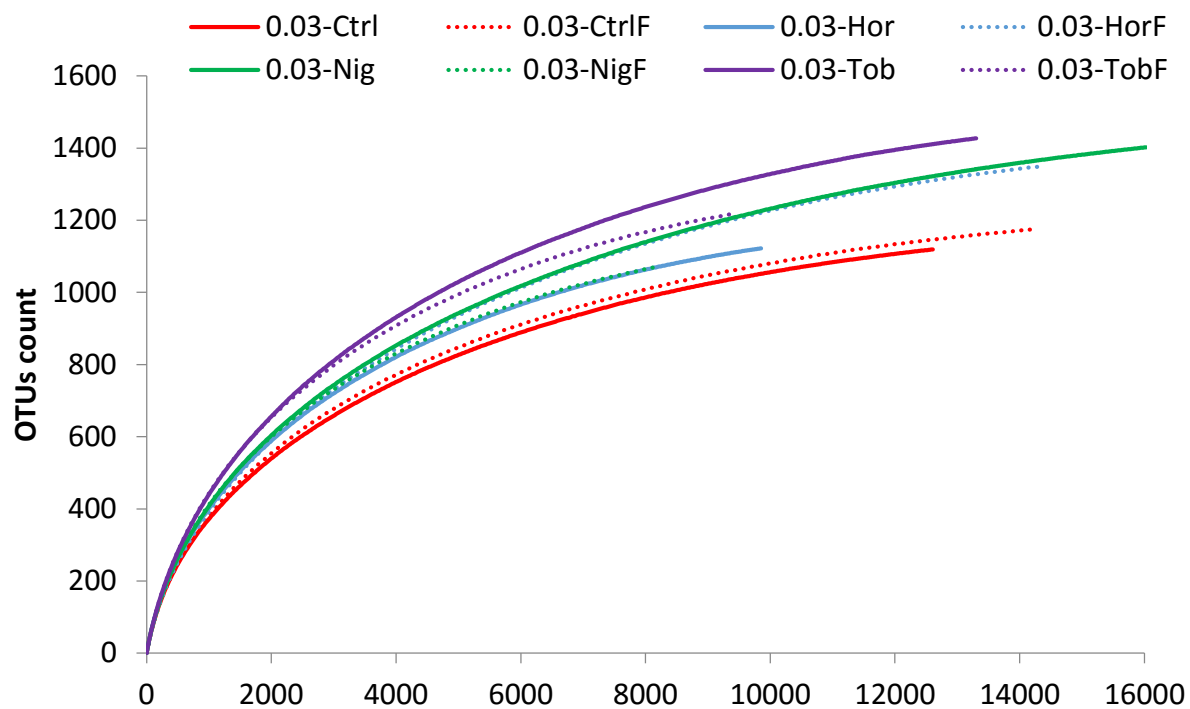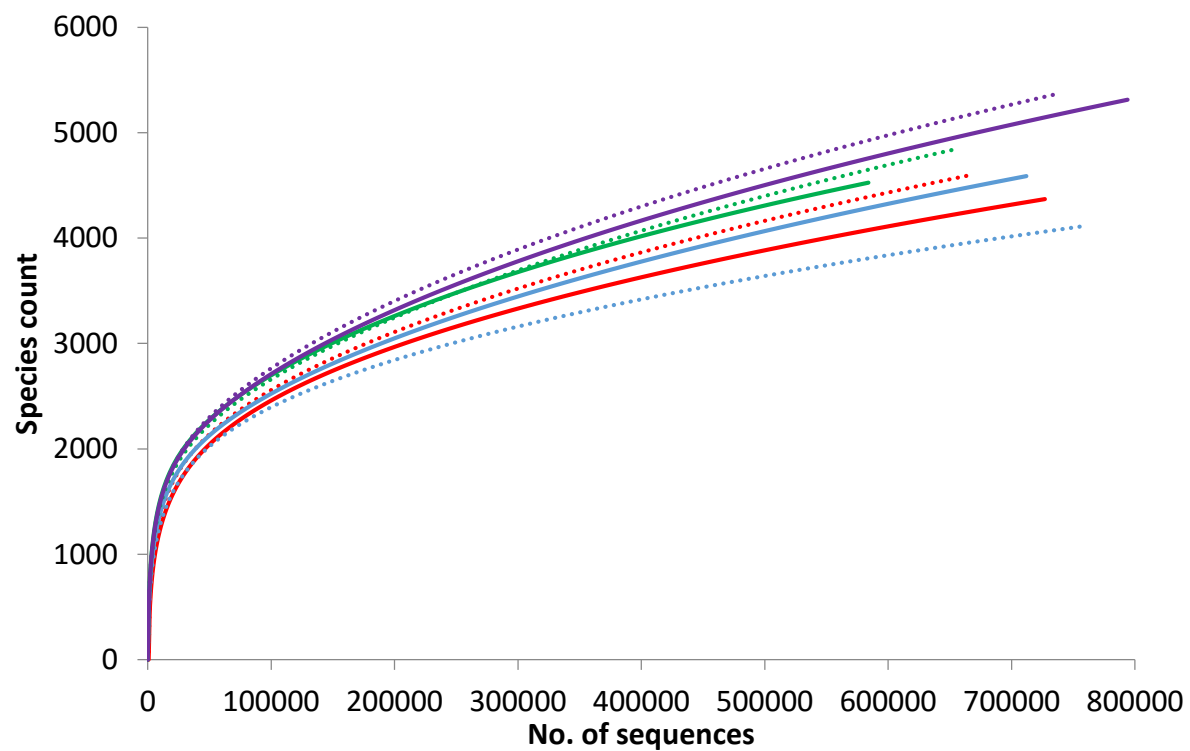

Supplement: FIGURE S1 — Overview of relative abundance of shotgun reads affiliated to COG categories. Abbreviations correspond to those in Figure 1. [file Data_Sheet_1.ZIP › Supplementary Figure 3.pdf]

**Supplementary Table 1:**


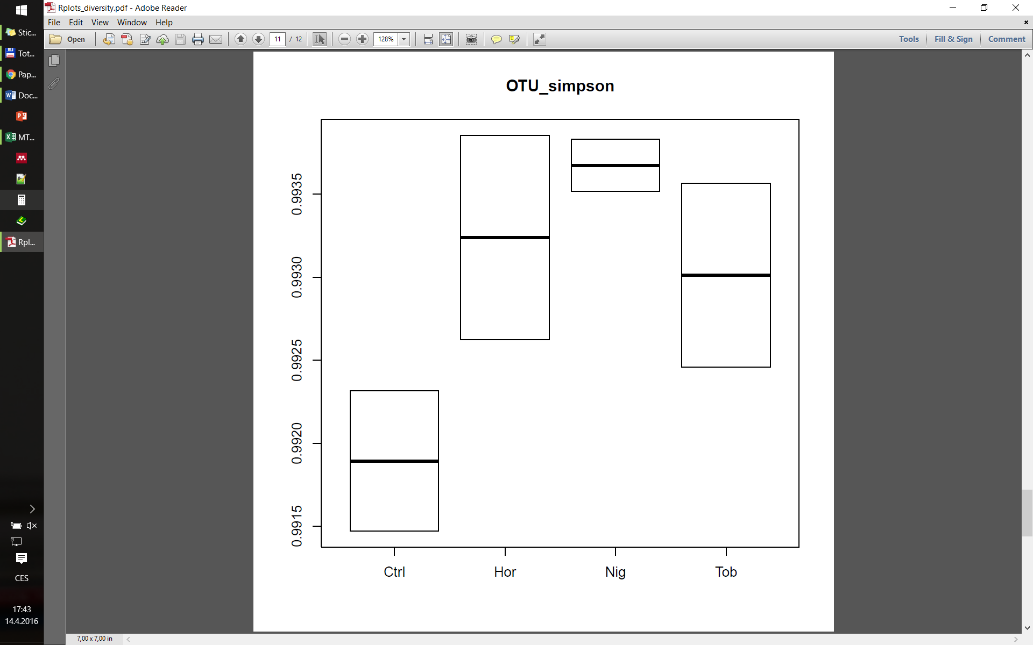


*λ*

| Sample | Simpson diversity index (*λ*) |
| --- | --- |
|
| Ctrl | 0.9923152 |
| CtrlF | 0.9914705 |
| Hor | 0.9938542 |
| HorF | 0.9926230 |
| Nig | 0.9935159 |
| NigF | 0.9938309 |
| Tob | 0.9924577 |
| TobF | 0.9935668 |

Supplement: FIGURE S1 — Overview of relative abundance of shotgun reads affiliated to COG categories. Abbreviations correspond to those in Figure 1. [file Data_Sheet_1.ZIP › Supplementary Table 1.docx]
